# The effectiveness of balneotherapy on pain, walking, and function in patients with diabetic neuropathy: a prospective observational study

**DOI:** 10.1007/s00484-024-02808-0

**Published:** 2025-01-08

**Authors:** Gabriela Dogaru, Alina Deniza Ciubean, Luminița Marinescu, Bianca-Maria Pop, Gabriel-Sorin Pașca, Lorena Ciumărnean

**Affiliations:** 1 Department of Medical Rehabilitation, “Iuliu Haţieganu” University of Medicine and Pharmacy Cluj-Napoca, Romania, Victor Babeş Str., No. 43, 400012 Cluj-Napoca, Romania; 2 Dept. of Rehabilitation Medicine, Clinical Rehabilitation Hospital, Viilor Str, No 46-50, 400066 Cluj-Napoca, Romania; 3 Geriatrics – Gerontology Discipline, Department of Medical Specialties, University of Medicine and Pharmacy “Iuliu Hatieganu”, Clinical Municipal Hospital, Cluj-Napoca, Romania; 4 Doctoral School of Biomedical Sciences, University of Oradea, 410073 Oradea, Romania; 5 5th Department of Internal Medicine, “Iuliu Haţieganu” University of Medicine and Pharmacy Cluj-Napoca,Romania, Victor Babeş Str., No. 43, 400012 Cluj-Napoca, Romania

**Keywords:** Carbonated mineral waters, Diabetes mellitus, Diabetic neuropathy, Medical rehabilitation, Physiotherapy, Physical exercises

## Abstract

The treatment of type 2 diabetes patients with diabetic neuropathy using pharmacological and non-pharmacological measures remains a current topic. The aim of this study is to evaluate the effect of comprehensive medical rehabilitation programs that include natural therapeutic factors (carbonated natural mineral water) on pain, gait, and functional status in these patients. Fifty patients diagnosed with type 2 diabetes and diabetic neuropathy in the lower limbs participated in the study. Half of them (DZ-PT) underwent treatment consisting of physical exercises, physiotherapy, and terrain cure, while the other half (DZ-CMW) received the same treatment plus baths with carbonated natural mineral water. Patients were evaluated using assessment scales for pain Visual Analogue Scale (VAS), the 10-meter walking test (W10m), lower limb muscle strength (FM), Functional Independence Measure (FIM), and Neuropathy Disability Score (NDS). The results showed a statistically significant reduction in pain assessed by VAS at the end of the treatment, persisting for three months (*p*-values < 0.001 between the two groups), with a greater reduction in the DZ-CMW group. Similarly, a significant improvement in gait, evaluated by W10m, was found both at the end of the treatment and at 3 months after its completion, with greater improvement in the DZ-CMW group. In conclusion, patients with type 2 diabetes with diabetic neuropathy can benefit from comprehensive medical rehabilitation programs periodically, including therapeutic natural factors, at balneoclimacteric resorts, alongside medication, dietary regimen, and physical activity.

## Introduction

In a world that promotes industrialization and sedentary lifestyles, there is a dramatic increase in metabolic pathology with a true “explosion” of diabetes cases. According to data provided by the International Diabetes Federation (IDF), the number of people diagnosed with diabetes worldwide in 2021 was 537 million, with an estimated increase to 592 million by 2030 [1]. Diabetes thus becomes a formidable enemy, not only due to the large number of affected individuals but especially because of the micro- and macrovascular complications it causes, impacting morbidity, mortality, quality of life, and increasing the cost of care for affected individuals. The microvascular complications of diabetes can be present from the time of diagnosis in type 2 diabetes. The affected organs include the retina (diabetic retinopathy), kidneys (diabetic nephropathy), and peripheral nerves (diabetic neuropathy) [2].

Diabetic neuropathy is one of the main complications of diabetes, affecting approximately 30% of diabetic patients throughout their lives [3]. The manifestations of the disease can vary depending on which part of the nervous system is affected (sensory/motor or autonomic), being one of the main causes of amputation in the elderly [4, 5]. Metabolic and inflammatory factors play an important role in the pathophysiology of diabetic polyneuropathy. Axonal degeneration with primary and secondary demyelination, or endothelial microangiopathy, has been demonstrated in biopsies taken from both animals and humans with diabetic polyneuropathy [6]. The main cause of diabetic neuropathy is hyperglycemia, but the pathogenic mechanisms are multiple [7, 8]: accumulation of sorbitol and fructose, structural damage to nerve membranes, vascular causes (nerve vessel damage), immune processes: anti-neuronal antibodies.

The treatment of diabetic peripheral neuropathy involves primarily treating the underlying disease, namely diabetes mellitus. In the specialized literature, there are no complex studies certifying a protocol for the rehabilitation treatment of diabetic peripheral neuropathy that includes balneotherapy or treatment with carbonated natural mineral water, alongside physiotherapy and physical exercises. According to the results of some studies, various forms of therapy have beneficial effects on diabetic peripheral neuropathy, especially on pain, such as transcutaneous electrical nerve stimulation (TENS), electromagnetic nerve stimulation with modulated frequencies (FREMS) [9], and magnetotherapy [10–12]. Balneotherapy involves treatment with gas molecules (CO_2_, H_2_S - mofettes, sulfides), or other natural factors, natural/mineral thermal waters, mud in numerous conditions [13–15]. A wide variety of indications for baths with carbonated mineral water (CO_2_) is mentioned in the specialized literature, including chronic circulatory disorders based on atherosclerotic diseases such as occlusive peripheral arterial disease, trophic ulcers, microangiopathies of various origins, and mild arterial hypertension [15]. Additionally, CO_2_ baths applied to an ischemic limb can induce local augmentation of vascular endothelial growth factor with the formation of NO-dependent neo-capillaries and associated mobilization of endothelial progenitor cells [16].

Type 2 diabetes mellitus (T2DM), formerly known as an adult disease, is on the rise with some peculiarities: in developed countries, new cases are largely represented by individuals over 65 years old, while in developing countries (e.g., Romania), the majority of cases occur between 45 and 65 years of age. Worldwide, there has been an increase in new cases of T2DM in children and adolescents, increasing the risk of chronic complications at younger ages [17]. Lifestyle modification through adopting appropriate nutrition programs, promoting physical exercise, and specific interventions from the prediabetes stage (impaired fasting glucose - IFG - or impaired glucose tolerance) can achieve primary prevention in T2DM. The Finnish Diabetes Prevention Study (DPS) was the first controlled, randomized study to examine the effect of a lifestyle modification intervention in preventing T2DM [18]. The Da Qing study [19] conducted in an Asian population (Chinese) with prediabetes (577 adults), with a mean age of 45 years, monitored for 6 years under diet and physical exercise, showed a 31% reduction in the risk of developing type 2 diabetes for those with nutritional intervention, a 46% reduction in the risk of developing diabetes for those with structured physical activity, and a 42% reduction in type 2 diabetes for those who followed both diet and physical exercise. Long-term follow-up of patients in the Da Qing study has demonstrated that preventing the onset of diabetes through lifestyle modification is associated with reduced overall and cardiovascular mortality after 23 years [20].

Overweight and obese individuals have a higher risk of developing type 2 diabetes, requiring structured behavioral therapy for weight loss and planned physical activity. Nutritional intervention should include limiting/eliminating the consumption of sugar and refined carbohydrates from the diet and replacing them with low glycemic index foods [21, 22], reducing portion sizes, adopting diets rich in fiber, with an emphasis on whole grains, legumes, and berries [23]. Additionally, nutritional recommendations aim to optimize vitamin D levels (optimal >/= 30 ng/ml) through oral supplements, with a daily intake of 2000–4000 units, as vitamin deficiency increases the risk of developing diabetes [24]. Consumption of fatty fish, cod liver, and sun exposure is encouraged to prevent/correct deficiency. Reducing refined, highly processed foods can contribute to reducing the risk of diabetes [25]. Physical inactivity and sedentary behavior are among the main public health issues [26].

Moderate-intensity physical activity practiced for 150 min/week (e.g., brisk walking) can improve insulin sensitivity and reduce abdominal fat [27]. Intense physical activity should be carefully supervised and adapted based on training and functional capacity [28].

At the European level, one of the effective ways to combat modern civilization diseases is offered by balneotherapy. The international trend is towards a return to nature for treatment and recreation. Additionally, climate changes, including alterations in climatic parameters, temperature, precipitation, changes in ultraviolet radiation levels, lead to air quality degradation, with a significant impact on human health. By increasing the intensity and duration of heatwaves, it is considered that there will be increases in the number of illnesses and deaths among cardiovascular patients and beyond [29].

Prolonged rest, reduced contact with nature, insufficient or lack of physical activity, carrying out activities in closed, air-conditioned spaces, generate deconditioning syndrome characterized by the impairment of thermoregulatory functional capacity correlated with immune, cardiovascular, respiratory, and muscular defense mechanisms (hypokinetic syndrome) and represent a risk factor in the onset of many diseases.

Therefore, the approach to patients with diabetes, by its nature, is holistic and multidisciplinary. Alongside diet, antidiabetic treatments, balneotherapy, through the therapeutic natural factors found in spa resorts, can be included in medical rehabilitation programs, alongside physiotherapy, physical activity, nature walks, and walking through the parks, thereby facilitating lifestyle changes.

The aim of this study was to evaluate the effect of the natural factor, carbonated mineral water, along with physical exercises, terrain cure, and physiotherapy on pain, gait, and function in patients with diabetic neuropathy admitted to the Baile Tusnad balneoclimatic resort, Romania, as well as the long-term effectiveness of these treatments at 3 months.

## Materials and methods

### Study design

This is a prospective, observational, longitudinal study conducted over a period of 14 months, from March 2022 to April 2023, in a balneoclimatic resort in Romania, Baile Tusnad.

### Subjects

The study included patients who came for medical rehabilitation treatment at the balneoclimatic resort of Băile Tușnad and were diagnosed with non-insulin-dependent type 2 diabetes and diabetic polyneuropathy for 4 to 15 years (sensory, sensorimotor) in the lower limbs and who are following the dietary and antidiabetic medication treatment recommended by the diabetologist. The study enrolled 50 patients aged between 55 and 75 years, who were hospitalized for 16 days at the Balneotherapy and Medical Rehabilitation Complex Băile Tușnad for balneotherapy-physio-kinetic treatment. The patients included in the study had been coming to the resort annually since being diagnosed with type 2 diabetes, once a year for medical rehabilitation treatment. Demographically, 24 patients were female (48%), 26 patients had sensory neuropathy (52%), and 24 had sensorimotor neuropathy (48%). The inclusion and exclusion criteria are summarized in Table 1. Written consent from the patients to participate in the study was obtained, and during the study, the patients did not receive treatments with analgesics, anti-inflammatories, or neurotropics.

**Table 1:** Inclusion and exclusion criteria

Inclusion criteria	Exclusion criteria
-Hospitalized at the Balneotherapy and Medical Rehabilitation Complex Băile Tușnad and coming annually to the resort. -Patient’s consent to participate in the study. -Conscious and cooperative. - Diagnosed with type 2 diabetes and diabetic polyneuropathy, following dietary and antidiabetic medication treatment recommended by the diabetologist. -Ability to undergo physiotherapy and balneotherapy treatments. -Patients will be included in the study regardless of previous administration of anti-inflammatory or analgesic medication. During the study, patients will not receive analgesic, anti-inflammatory, or neurotropic treatments. -Patients should be metabolically and hemodynamically stable.	-Non-cooperative patients. -Contraindications to physiotherapy and balneotherapy treatments. - Cardiac rhythm disorders - PVC (Premature Ventricular Contractions), AF (Atrial Fibrillation) - Uncontrolled blood pressure (with medication > 140/90 mmHg) - Acute dermatological conditions - Epilepsy - Hip/knee endoprostheses

### Outcome measurements

The patients were assessed using evaluation scales for pain Visual Analog Scale (VAS), the 10-meter walking test (W10m), lower limb muscle strength (MS), Functional Independence Measure (FIM), and Neuropathy Disability Score (NDS), both at admission and discharge, to observe the impact of the complex medical rehabilitation and balneology treatment on diabetic polyneuropathy. The VAS and W10m scales were also evaluated at 3 months after the completion of treatment to assess the long-term effects.

Out of the total number of patients hospitalized between March 2022 and April 2023 and diagnosed with non-insulin-dependent type 2 diabetes and diabetic polyneuropathy (sensory, sensorimotor) in the lower limbs, only 50 met the inclusion criteria and were included in the study. One group (DZ-PT), consisting of 25 patients, of which 12 (48%) had sensory neuropathy and 13 (52%) had sensorimotor neuropathy, underwent a 14-day treatment regimen. This regimen included daily sessions of 30 min of physical exercises, treatment with artificial physical agents (physiotherapy), specifically unipolar galvanic baths for the lower limbs with analgesic and vasodilator effects for 10 min/day, transcutaneous electrical nerve stimulation (TENS) for neuropathic pain inhibition for 10 min/day, and outdoor therapy for 30 min/day. The second group (DZ-CMW), comprising 25 patients, 14 (56%) with sensory neuropathy and 11 (44%) with sensorimotor neuropathy, underwent the same treatment regimen with the addition of daily treatment with specific natural physical agents from the Băile Tușnad balneological resort, namely, baths with natural carbonated mineral water (32 °C) for 20 min/day, the who wished to, respectively, and those who did not have contraindications to these baths (some cardiac rhythm disorders - PVCs, medically uncontrolled hypertension, some with subacute dermatological conditions). Of course, coming annually, patients in the T2DM-PT group had previously undergone treatment with carbonated mineral waters, but at the time of inclusion in the study, they did not receive this treatment for the reasons mentioned earlier. These medical rehabilitation programs are recommended after a prior consultation. Treatments were administered at the recommendation of the medical rehabilitation doctor, under the guidance and supervision of the rehabilitation assistant. The same treatment objectives were adhered to for each patient included in the study to achieve a similar functional impact.

The exercise program included moderately intense aerobic exercises (heart rate 50–70% of maximum heart rate), anaerobic resistance exercises (with weights), balance exercises, muscle toning exercises, and walking exercises. Outdoor therapy (climatotherapy) involved walking, light jogging on a standardized route in terms of length, elevation difference, walking pace, aimed at training the cardiovascular system, improving walking, increasing neuromuscular tone, improving venous and arterial circulation, and joint mobility. Carbonated mineral water was used from springs 7 and 8, with a total mineralization of 122.036 mmol/L. The composition of the carbonated mineral water was as follows: chlorine 49.999 mmol/L, bromine 0.020 mmol/L, sulphates 0.0666 mmol/L, HCO3-13.000 mmol/L, sodium 44.161 mmol/L, potassium 2.307 mmol/L, calcium 4.698 mmol/L, magnesium 3.088 mmol/L, iron 0.376 mmol/L, carbon dioxide 27.000 mmol/L.

The subjects’ pain was evaluated using the Visual Analog Scale (VAS), where pain intensity ranged from 0 to 10, with 0 representing no pain and 10 representing unbearable pain, before the start of treatment (day 1), at the end of treatment (day 14), and at three months after its completion (month 3). Patients were contacted by phone for pain assessment by the medical rehabilitation doctor using the same scale.

On days 1 and 14, before and after treatment, the 10-meter walking test (W-10 m) was conducted [30]. Patients were asked to walk at a comfortable speed, and the time to cover the 10-meter distance was recorded. The test involved walking in one direction without turning back. There are no limitations for daily activities if the time is equal to or less than 10 s. Between 10 s and less than or equal to 20 s, reduced mobility with functional limitation is considered, while less than 30 s indicates severe mobility limitation requiring assistance. At discharge, patients received recommendations to continue physical exercises and walking at home. Patients were contacted by phone at three months for gait evaluation, which was performed by the patient.

On days 1 and 14, muscle strength (MS) was measured for the lower limbs using a grading scale from 1 to 5, where 0 indicates total paralysis, 1 indicates probable or visible muscle contraction but ineffective, 2 indicates unsatisfactory contraction with full range of motion if gravity is eliminated, 3 indicates satisfactory contraction with full range of motion against gravity, 4 indicates good contraction with full range of motion against gravity and moderate resistance, and 5 indicates normal contraction with normal range of motion and strength within age and sex limits [30].

Additionally, the Functional Independence Measure (FIM) was assessed before treatment (day 1) and at two weeks (day 14) after treatment completion. Scores of 7 (complete independence) and 6 (modified independence) indicate that subjects do not require assistance. Subjects require assistance if they have Modified Dependence-5 (supervision), 4 (minimal assistance), 3 (moderate assistance), 2 (maximal assistance), or 1 (total assistance) [30].

On days 1 and 14, Neuropathy Disability Score (NDS) [26] was measured and calculated for both the right and left lower limbs, respectively, for pin-prick (normal = 0, abnormal = 1) and Achilles reflex (present = 0, present with reinforcement = 1, absent = 2).

MS, FIM, and NDS scales could not be performed at 3 months as they require the physical presence of patients, who did not return to the resort.

**Fig. 1:**
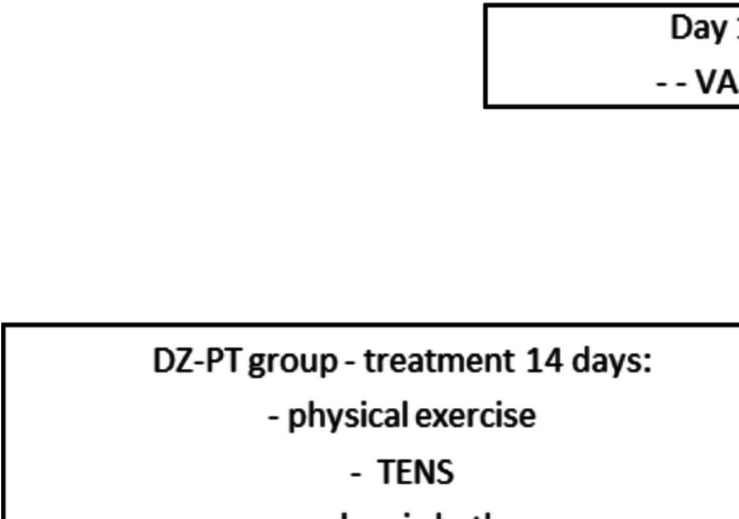
Flowchart of the study protocol.*VAS Visual Analogue Scale, *W-10 m *the 10-meter walking test, *MS *lower limb muscle strength, *FIM *Functional Independence Measure, *NDS *Neuropathy Disability Score, *DZ-PT *type 2 diabetes and physical therapy, *DZ-CMW *type 2 diabetes and carbonated mineral waters, *TENS *transcutaneous electrical nerve stimulation

The study protocol, flowcharted in Fig. 1, was designed in accordance with the principles of the Declaration of Human Rights and was approved by the Ethics Committee of the Băile Tușnad Balneotherapy Complex (approval no. 2/ February 17, 2022).

### Data analysis

We performed statistical analysis using Jasp 0.18.3.0. When the quantitative data were found not to follow the normal distribution, we reported them as the median and interquartile range, defined as [Q1 to Q3] (the value of the first – Q1 and third – Q3 quartile). We reported the mean and standard deviation for data that violated normal distribution but had equal values for Q1 and Q3. Qualitative data were reported as number and percentage. Comparison between two independent groups was made with Chi-squared or Fisher’s exact test according to the expected frequencies for qualitative data and Mann Whitney test for quantitative data. Paired repeated measurements were tested with Wilcoxon signed ranks. Repeated measurements of scores evaluated more than twice were compared on each group with Friedman’s test followed by Conover’s post-hoc analysis in case of statistically significant differences. Raincloud plots were used to visually represent the distribution of scores within each group on evaluations at different times. The significance level for the statistical analysis was set at 5%, and we considered results to be statistically significant if the *p*-values were smaller than 0.05.

## Results

Fifty patients, aged between 55 and 75 years old, with 25 subjects in each group, were included in the study. In our study, all patients managed to complete the recommended treatment.

Half of the women were under 64 years old, while half of the men were under 68.5 years old (Mann Whitney test: *p*-value = 0.031). The majority of women had sensory neuropathy (17/26), while most men had sensorimotor neuropathy (17/24) (Chi-squared test: *p*-value = 0.010).

**Fig. 2:**
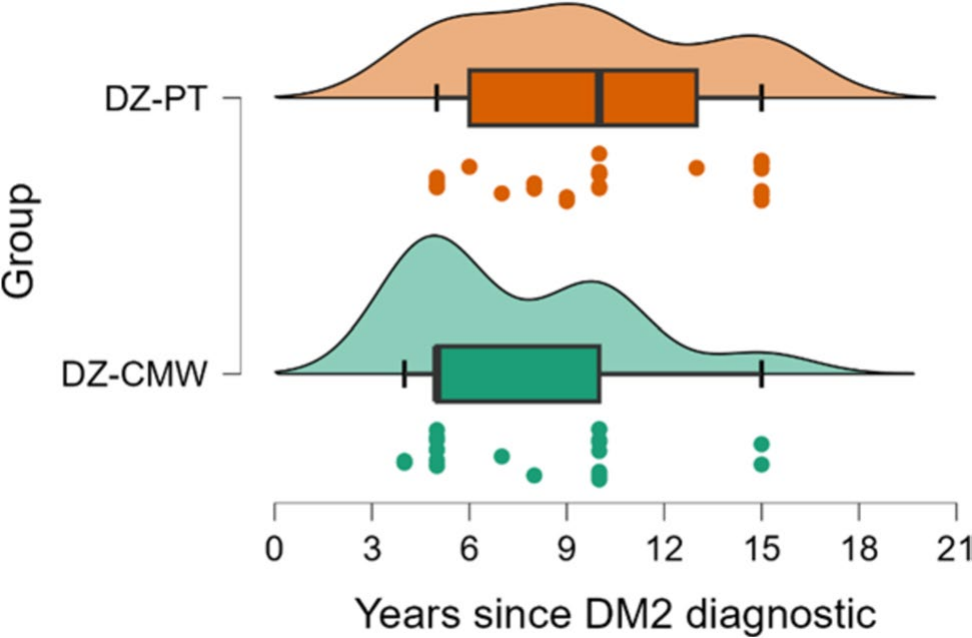
Time expressed in years since living with DM2

The subjects were similar between groups regarding demographic characteristics (Table 2), except for the onset of type 2 diabetes (Fig. 2). Patients in the DZ-PT group had a median of 10 years [6 to 13] since being diagnosed with type 2 diabetes, compared to the DZ-CMW group with 5 years [5 to 10], with a* p*-value of 0.042.

**Table 2:** The demographics of subjects by groups

Characteristic	All, *n*=50	DZ-CMW, *n*=25	DZ-PT, *n*=25	*p*-value
Age, years ^a^	67.5 [63 - 70]	68 [63 - 70]	67 [64 - 70]	0.915
Sex, women ^b^	24 (48)	12 (48)	12 (48)	>0.999
Neuropathy ^b^				0.571
Sensory	26 (52)	14 (56)	12 (48)	
Sensory-motor	24 (48)	11 (44)	13 (52)	
DM 2 onset, years ^a^	8.5 [5 to 10]	5 [5 to 10]	10 [6 to 13]	0.042

^a^ results are expressed as median [Q1 to Q3] where Q is the quartile; comparison between groups with Mann Whitney test; ^b^ results are expressed as number (%) and comparison between groups was made with Chi-squared test

At the study inclusion, patients had muscle strength (MS) in the lower limbs with values of 4 and 5, and were functionally independent with scores of 7 and 6, with only one patient scoring 5, indicating the need for supervision, on the Functional Independence Measure (FIM) scale. All patients underwent annual medical rehabilitation treatments since being diagnosed with type 2 diabetes.

**Fig. 3:**
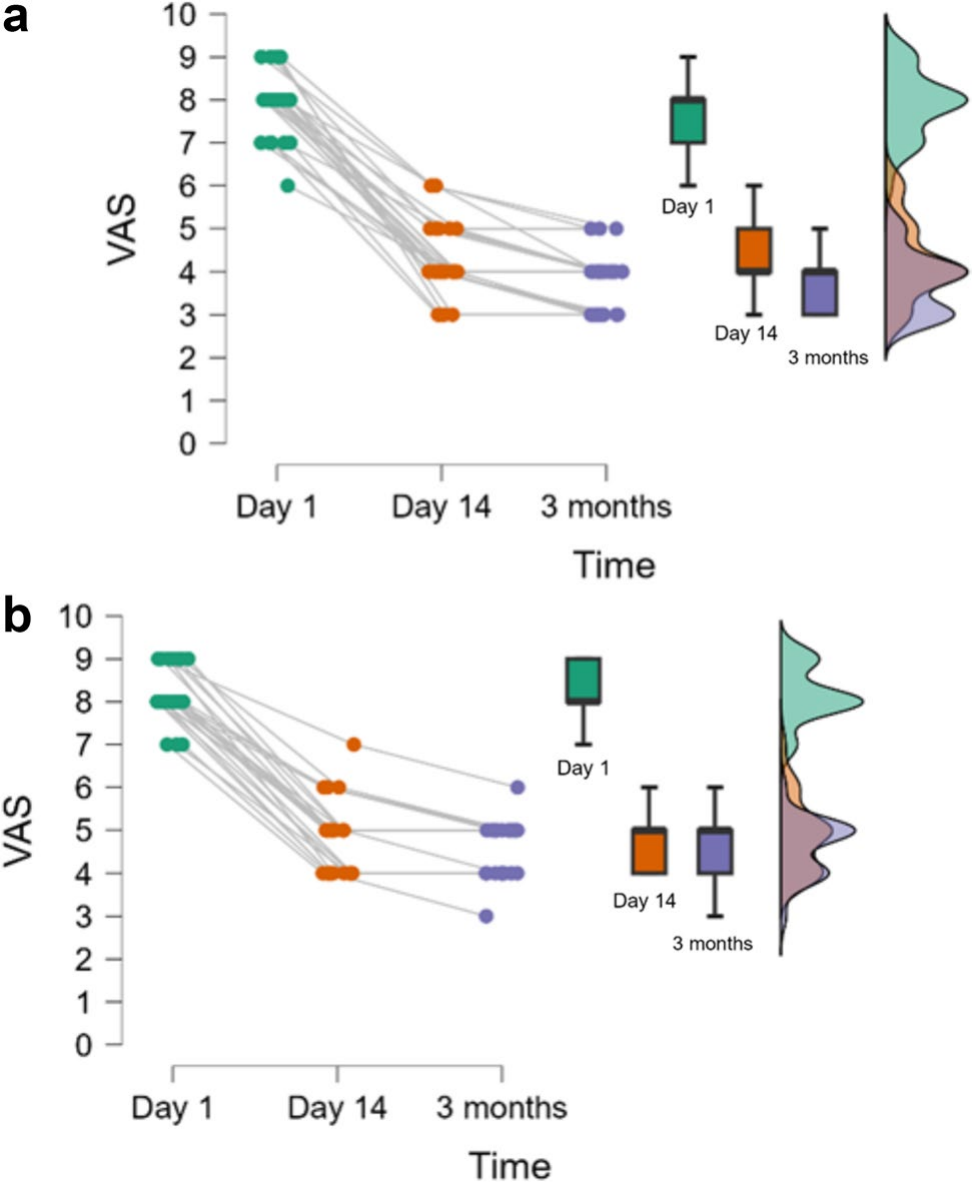
**a** Distribution of VAS in time for DZ-CMW (< 0.001 for Day 1 vs. Day 14 and Day 1 vs. 3 months & 0.055 for Day 14 vs. 3 months); **b** Distribution of VAS in time for DZ-PT (< 0.001 for Day 1 vs. Day 14 and Day 1 vs. 3 months & 0.278 for Day 14 vs. 3 months)

**Fig. 4:**
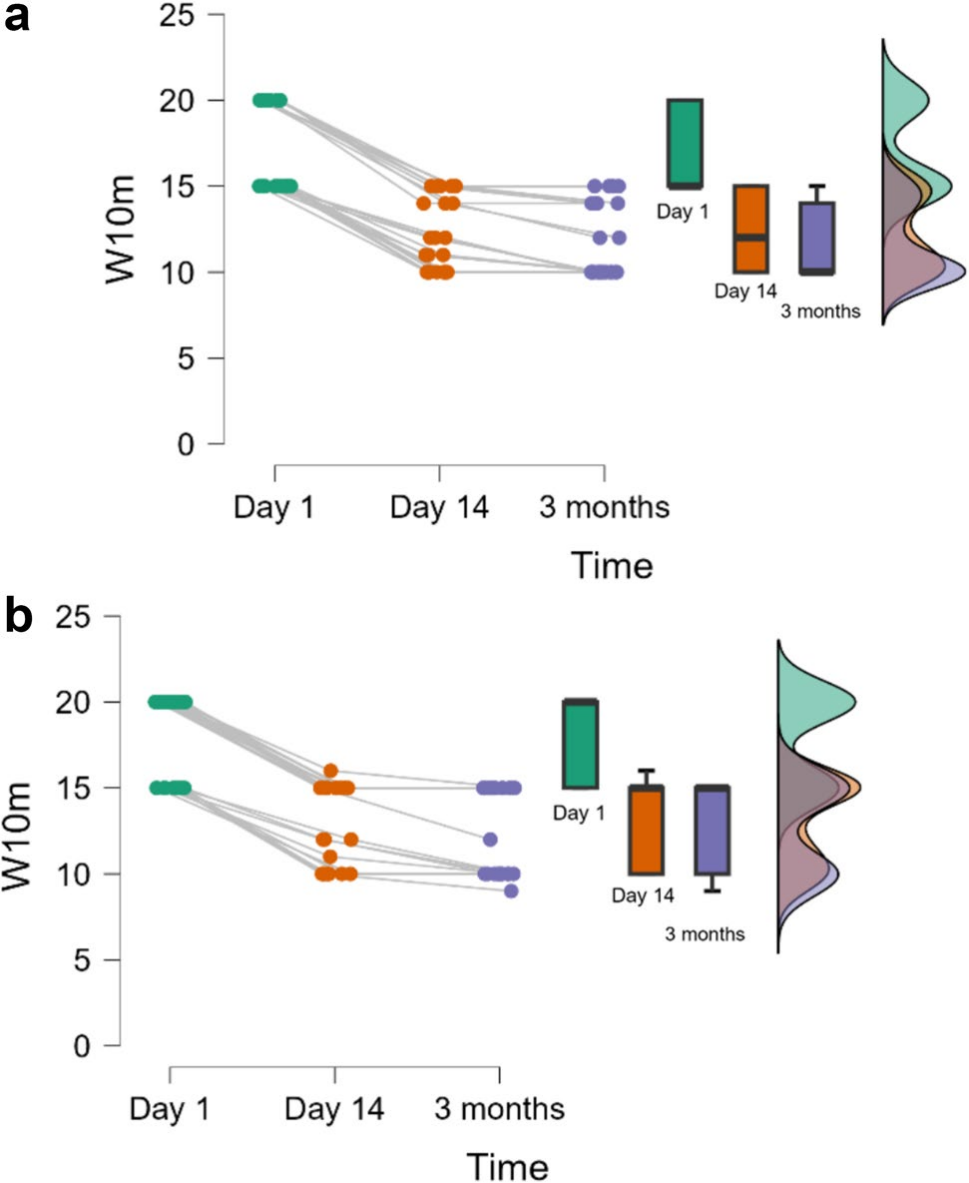
**a** Distribution of W10m in time for DZ-CMW (< 0.001 for Day 1 vs. Day 14 and Day 1 vs. 3 months & 0.099 for Day 14 vs. 3 months); **b** Distribution of W10m in time for DZ-PT (< 0.001 for Day 1 vs. Day 14 and Day 1 vs. 3 months & 0.278 for Day 14 vs. 3 months)

Only the pain score at 3 months proved statistically significantly different between groups (Table 3), with statistically significant improvements in VAS and W10m scores within the same group (Figs. 3 and 4). No changes in short-term follow-up were observed for MS, FMI, NDSL, and NDSR (Table 3).

**Table 3:** Measurements by group

Characteristic	All, *n*=50	DZ-CMW, *n*=25	DZ-PT, *n*=25	*p*-value*
VAS				
Day 1	8 (0.7)	7.9 (0.8)	8.2 (0.6)	0.202
Day 14	4.6 (0.9)	4.3 (0.9)	4.9 (0.8)	*0.017*
3 months	4.2 (0.8)	3.7 (0.7)	4.6 (0.6)	***<0.001***
*p*-value **	***<0.001***	***<0.001***	***<0.001***	
W10m				
Day 1	17.4 (2.5)	17 (2.5)	17.8 (2.5)	0.267
Day 14	12.7 (2.3)	12.2 (2.2)	13.1 (2.3)	0.130
3 months	12.1 (2.4)	11.6 (2.1)	12.6 (2.5)	0.102
*p*-value **	***<0.001***	***<0.001***	***<0.001***	
MS				n/a
Day 1 & Day14				
4	13 (26)	5 (20)	8 (32)	
5	37 (74)	20 (80)	17 (68)	
FMI				n/a
Day 1 & Day 14				
5	1 (2)	0 (0)	1 (4)	
6	10 (20)	6 (24)	4 (16)	
7	39 (78)	19 (76)	20 (80)	
NDSL				n/a
Day 1 & Day 14				
0	6 (12)	3 (12)	3 (12)	
1	29 (58)	14 (56)	15 (60)	
2	15 (30)	8 (32)	7 (28)	
NDSR				n/a
Day 1 & Day 14				
0	6 (12)	3 (12)	3 (12)	
1	29 (58)	14 (56)	15 (60)	
2	15 (30)	8 (32)	7 (28)	

* Mann Whitney test; ** Friedman test; *n/a* = not applicable

In both the DZ-PT and DZ-CMW groups, an improvement in pain was observed at 14 days (4.9(0.8) versus 4.3(0.9) from treatment), as well as at three months (4.6(0.6) versus 3.7(0.7)), statistically significant. A greater improvement in pain was observed in the DZ-CMW group, statistically significant with *p* < 0.001.

The evaluation of W10m showed an improvement in walking at 14 days and at three months in both groups, statistically significant. However, there was no statistical significance when comparing between groups (*p* = 0.102). Nevertheless, the values obtained indicate a greater improvement in walking quality in the DZ-CMW group.

There were no changes between day 1 and day 14 in the parameters of MS, FMI, NDSL, and NDSR, over a relatively short period. However, it is important to note that muscle strength and functional independence were maintained through annual continuation of balneotherapy and medical rehabilitation treatments (Table 3). Only 13(26) patients had a muscle strength of 4, with the remaining 37(74) scoring 5, both on day 1 and day 14. Additionally, only one patient from the DZ-PT group had a score of 4 on the FMI scale, indicating the need for walking supervision, while the rest were independent. The score values remained the same for both groups at 14 days of treatment on the NDSL and NDSR scales.

## Discussion

In this study, we aimed to evaluate the effects of medical rehabilitation treatments in the balneoclimatic resort of Baile Tusnad, Romania, which includes carbonated mineral water baths (CO_2_), on patients diagnosed with non-insulin-dependent type 2 diabetes and diabetic neuropathy of the lower limbs, along with physical exercises, terrain cure, and physiotherapy consisting of TENS application and galvanic baths for the lower limbs. We observed a statistically significant improvement in pain in both groups at the end of the treatment, at 14 days (DZ-PT 4.9, DZ-CMW 4.3), as well as at the 3-month evaluation (DZ-PT 4.6, DZ-CMW 3.7). The values suggest a greater reduction in pain in the DZ-CMW group, which also underwent carbonated mineral water baths as part of the treatment. Additionally, a statistically significant improvement in walking distance (W10m) was observed in both groups at 14 days of treatment (DZ-PT 13.1, DZ-CMW 12.2) and at the 3-month evaluation (DZ-PT 12.6, DZ-CMW 11.6), with a more efficient improvement in those who received carbonated mineral water. However, we must consider that patients in the DZ-PT group, according to statistical analysis, had an average of 10 years [6 to 13] since being diagnosed with diabetes, compared to the DZ-CMW group, who had an average of 5 years [5 to 10], with a *p*-value of 0.042. During this period, these patients came to the resort annually for balneotherapy treatments. Pain improved in both groups after 14 days and 3 months of treatment, with statistically significant results. However, at 3 months, the VAS score in the DZ-CMW group treated with carbonated mineral water was lower, at 3.7 (0.7), compared to the DZ-PT group, which had a score of 4.6 (0.6), a statistically significant difference. This suggests a more prolonged beneficial effect of the treatment.

After discharge, patients received recommendations to continue the physical exercises performed during treatment, to be continued at home for 20 min/day, as well as to walk for at least 10 min/day.

Muscle strength (MS), Functional Independence Measure (FMI), Neuropathy Disability Score (NDSL), and Neuropathy Disability Score Right (NDSR) were not altered at the end of treatment, receiving the recommendation to undergo annual medical rehabilitation treatments, including natural therapeutic factors.

Results of some studies have shown that the beneficial and long-lasting effects of CO_2_ baths, as well as of balneotherapy treatments, can be achieved only through serial applications, which determine their final efficacy. These medium and long-term effects are not only the sum of individual effects but rather are brought about by fundamental changes in the autonomic nervous system regarding stimulation, response, and adaptive therapy, which require further ongoing studies [16, 31, 32]. The objective of a study conducted on a sample of 394 patients at Baile Tusnad was to investigate patients’ perception of the quality and effectiveness of balneotherapy treatments, especially since many patients undergo treatment annually or even twice a year [33]. The results showed a duration of over a month, with the maximum frequency of those who feel the effect for a duration of 2–6 months after leaving the resort (54–62%), and approximately one-third of patients (33%) stated that the effect of treatments lasts more than six months. Also, for patients who regularly undergo treatment (once or twice a year), the high frequency of long-term effects (more than 6 months) is maintained even in older age groups (71–80 years and over 80 years) [33]. Also in this study [33], the clinical symptom that improved most noticeably following balneotherapy and physiotherapy treatments was tracked. The majority of patients (55–61%) reported pain relief, both among those who regularly visit the resort and those who come occasionally. Secondly, patients observed an improvement in quality of life by 22–24% (without differentiation based on frequency of resort visits). Improvement in gait quality and/or increased functional independence was reported by a considerably smaller number of patients (9–10% and 5%, respectively) compared to the previously mentioned symptoms. The study on the effect of carbonated mineral water from spring number 7 at Baile Tusnad on the oxidative stress balance/antioxidant parameters in plasma, associated with experimental myocardial ischemia in rats, resulted in a reduction in total oxidative stress parameters, which are important contributors to ischemia progression and lesion amplification, and improvement in antioxidant status, which is an essential beneficial factor in limiting the ischemic process [34].

The therapeutic approach to patients with diabetes mellitus should include diet, pharmacological treatment, physical activity, and medical rehabilitation treatments. The American Diabetes Association (ADA) guidelines recommend a minimum of 150 min of moderate to vigorous physical activity (50–70% maximum heart rate) per week for patients with type 2 diabetes, including aerobic exercises (running, swimming, cycling, brisk walking), resistance exercises (weight training), and balance exercises. Studies show that during physical exertion, blood glucose levels decrease, and after stopping physical activity, blood glucose continues to decrease, with glucose being used to replenish glycogen reserves. Maintaining normal blood glucose levels (necessary for brain function) is achieved by rapid release of glucose from muscle and liver glycogen stores, and counterregulatory hormones (glucagon, cortisol, growth hormones) are also released, inducing gluconeogenesis and glycogenolysis to maintain normoglycemia [1].

There are numerous studies on small patient cohorts that have shown the favorable effect of various physiotherapy procedures in diabetic peripheral neuropathy. Pulsed frequency magnetotherapy in studies has increased nerve conduction velocity, increased muscle action potential amplitude, and increased number of motor units. High-frequency transcutaneous electrical nerve stimulation (TENS) reduces neuropathic pain in diabetic peripheral neuropathy by inhibiting the excitability of the sensory nervous system. Application of galvanic baths, low-frequency current, causes vasodilation and analgesia, with action on sensory and motor nerve fibers [10, 11, 35].

There are also studies showing that series of baths in CO_2_-enriched mineral water can influence the activity of skin thermoreceptors [36], possibly beneficial in sensory disorders within diabetic neuropathy. Balneotherapy treatments, including hydrotherapy, have been and continue to be studied in pain relief in various pathologies [37, 38].

Immersion in CO2-enriched water is considered to stimulate parasympathetic nerve activity in humans [39]. The effect of CO_2_-enriched water on subcutaneous microcirculation may be caused by peripheral vasodilation due to increased parasympathetic activity and decreased sympathetic activity, and artificial CO_2_ foot baths are clinically effective in saving ischemic limbs [36]. In animal experiments, applying a CO_2_ bath has an effect on experimentally induced inflammation. Results showed that carbonated water to which 3% NaCl was added provides an anti-inflammatory effect. According to data from a study, increased free radical neutralization activity is beneficial in occlusive arterial diseases, and this activity may reduce the systemic and local inflammatory response observed after ischemia-reperfusion injuries; the neutralizing agent could be carbonated mineral water baths [40].

In our study, we used natural carbonated mineral water, with a total mineralization of 122.036 mmol/L, whose composition according to laboratory data includes chlorine 49.999 mmol/L, sulphates 0.0666 mmol/L, HCO3-13.000 mmol/L, sodium 44.161 mmol/L, calcium 4.698 mmol/L, magnesium 3.088 mmol/L, and carbon dioxide 27.000 mmol/L. This suggests that the overall picture regarding the chemical constituents in a therapeutic spring should always be kept in mind.

Romania is among the European countries with remarkable spa and health potential. Approximately one-third of Europe’s mineral and thermal waters are found here, utilized in the prevention and medical recovery of many pathologies, including patients with type 2 diabetes and diabetic neuropathy. There is a trend towards integrating balneotherapy into a broader concept of holistic patient care. New medical rehabilitation programs should include, in addition to diet, physical activity, physiotherapy, balneotherapy, nature through spa parks, and behavioral education. For centuries, forests have provided benefits to the population and are vital for biodiversity, climate, human health, and the planet. However, a new report from the European Joint Research Centre shows that currently, only 3% of Europe’s forested area consists of primary/old-growth forests. Current literature supports the benefits of exposure to nature and green environments, specifically regarding the practice of forest bathing, for human health, including the function of the immune system, cardiovascular system (hypertension, coronary artery disease), respiratory system (allergies and respiratory diseases), diabetes, depression, and anxiety. Research conducted in Japan and China has highlighted a multitude of physiological and psychological benefits associated with Shinrin-Yoku (SY), known as forest bathing. There are studies involving adults diagnosed with type 2 diabetes, where it has been found that blood glucose levels decreased after multiple SY sessions, indicating a significant correlation between SY and blood glucose reduction [41, 42].

In primary care, incorporating physical activity into general weight loss and diabetes risk reduction programs has the potential to save costs and time [43, 44] Alongside nutrition and exercise, smoking cessation is also essential for preventing type 2 diabetes in known smokers. The risk of developing type 2 diabetes in smokers is approximately 44% and even over 60% in those who smoke more than 20 cigarettes/day. After quitting smoking for 5 years, the risk of developing diabetes decreases by 13%.

After the diagnosis of type 2 diabetes, the therapeutic plan should also focus on lifestyle management to achieve secondary and tertiary prevention, pharmacological therapy for diabetes and its complications, associated therapy for comorbidities involving other specialists where necessary, and psychosocial care with periodic evaluation of affective disorders, eating behavior, and cognitive function, especially in individuals over 65 years old diagnosed with diabetes [45].

Interdisciplinary collaboration is recommended for a comprehensive, holistic approach to diabetes and frequently associated pathologies in the form of cardiovascular, renal, ophthalmological, neurological, and digestive complications.

The current study has several limitations The lack of a control group may be considered one, but in balneotherapy complexes, each patient receives treatment. Another limitation is that no studies were found in the literature regarding patients with type 2 diabetes complicated by diabetic neuropathy and complex balneotherapy treatments, including baths with natural carbonated mineral water. Additionally, it would be useful in the future to monitor the effects of balneotherapy in the longer term. Another limitation of this study is that the quality of life or lifestyle changes were not quantified. However, for the modern evaluation of health, well-being, and quality of life, it is essential to continue and develop research activity in future studies, linked to establishing the mechanisms of action and effects of balneotherapy, alongside patient education regarding lifestyle.

## Conclusions

In conclusion, patients with type 2 diabetes and diabetic neuropathy can benefit from comprehensive medical rehabilitation programs periodically, including therapeutic natural factors, at balneoclimacteric resorts, alongside medication, dietary regimen, and physical activity.

The results showed a statistically significant reduction in pain, assessed by VAS, at the end of the treatment, persisting for three months (*p*-values < 0.001 between the two groups). Similarly, a significant improvement in walking, assessed by W10m, was found both at the end of the treatment and at 3 months after its completion. The holistic integration of pharmacological approaches, diet, medical rehabilitation, balneotherapy, and therapeutic education needs to be further analyzed to identify optimal synergies for restoring functionality, improving quality of life, and mitigating the long-term consequences of patients with type 2 diabetes with diabetic neuropathy.
